# Efficiency of fluorodeoxyglucose positron emission tomography/computed tomography to predict prognosis in breast cancer patients received neoadjuvant chemotherapy

**DOI:** 10.1186/s40064-015-1634-y

**Published:** 2015-12-24

**Authors:** Toshiyuki Ishiba, Tsuyoshi Nakagawa, Takanobu Sato, Makoto Nagahara, Goshi Oda, Hitoshi Sugimoto, Mai Kasahara, Tokuko Hosoya, Kazunori Kubota, Tomoyuki Fujioka, Peter Danenberg, Kathleen Danenberg, Hiroyuki Uetake

**Affiliations:** Department of Breast Surgery, Tokyo Medical and Dental University (TMDU), 1-5-45, Yushima, Bunkyo-ku, Tokyo, 113-8510 Japan; Liquid Genomics, Inc., 1725 Del Amo Blvd. Torrance, Torrance, 90501 CA USA; Department of Biochemistry and Molecular Biology, University of Southern California/Norris Comprehensive Cancer Center, 1975 Zonal Ave, Los Angeles, 90089-9151 CA USA; Department of Radiology, Tokyo Medical and Dental University (TMDU), 1-5-45, Yushima, Bunkyo-ku, Tokyo, 113-8510 Japan; Department of Surgical Specialities, Tokyo Medical and Dental University (TMDU), 1-5-45, Yushima, Bunkyo-ku, Tokyo, 113-8510 Japan

**Keywords:** FDG-PET/CT, Metastases, Breast cancer, Neoadjuvant chemotherapy, Prognostic marker

## Abstract

Neoadjuvant chemotherapy (NAC) has become a standard therapy for patients with advanced breast cancer. Pathological complete response (pCR) after NAC is an important prognostic indicator, but some patients with pCR continue to experience recurrence. So new predictive and prognostic markers in addition to pCR are needed following NAC for breast cancer. Fluorodeoxyglucose positron emission tomography/computed tomography (FDG-PET/CT) can evaluate metastases in the entire body simultaneously, and has several potential advantages over conventional imaging modalities. The purpose of this study was to evaluate whether FDG-PET/CT can determine NAC response and whether FDG-PET/CT can be a new prognostic marker. We imaged 83 breast cancer tumors with FDG-PET/CT, ultrasound (US), and magnetic resonance imaging (MRI) to evaluate NAC efficacy. As we previously analyzed 110 breast cancers with FDG PET/CT, we defined a threshold of >1.7 maximum standardized uptake value (SUV_max_) as abnormal fluorodeoxyglucose (FDG) uptake. After NAC, 16 (19.3 %) tumors had a complete response, 54 (65.1 %) had a partial response, 11 (13.3 %) showed stable disease, and 2 (2.4 %) showed progressive disease. One of the two patients with progressive disease had bone metastasis detected by FDG-PET/CT and was not operated on. Remote metastases were evident in 2.4 % of patients after NAC as determined by FDG-PET/CT. Overall, 17 patients had pathological complete response (pCR). The sensitivity of abnormal FDG uptake after NAC for non-pCR was 20.3 % and the specificity was 94.7 %. Patients with abnormal FDG uptake after NAC experienced significantly more recurrences (*P* = 0.004) and more of them died (*P* = 0.010). Moreover, the difference in disease-free survival was more significant in the estrogen receptor (ER)-negative group. FDG-PET after NAC may be more effective for predicting prognosis than for evaluating treatment response. This tendency was particularly remarkable in ER-negative breast cancer tumors. FDG-PET/CT is useful for reevaluating surgical applicability after NAC.

## Background

Neoadjuvant chemotherapy (NAC) has become a standard therapy for patients with locally advanced breast cancer or many lymph node metastases. NAC is useful for increasing the rate of breast-conserving surgery, treating minimum metastases, which may cause patient death, predicting patient prognosis, and for evaluating tumor treatment response (Fisher et al. 1998; Chollet et al. 1997; Rastogi et al. 2008; Vinnicombe et al. 1996; Abraham et al. 1996). Recent studies that investigated the prognostic effects of clinicopathologic factors in breast cancer patients with NAC have suggested that pathological complete response (pCR) is an important prognostic indicator (Chaturvedi et al. 2005; Kuerer et al. 1999; Buzdar et al. 2005). However, some patients with pCR continue to experience recurrences (Gonzalez-Angulo et al. 2005). Furthermore, many patients with non-pCR experience a good prognosis. Therefore, new predictive and prognostic markers in addition to pCR are sought following NAC for breast cancer.

Ultrasonography (US) and magnetic resonance imaging (MRI) have been widely used for measuring tumor size to determine NAC response. As breast cancers often metastasize to the liver, lymph nodes, lung, and bone, this disease generally requires multiple imaging modalities, such as enhanced computed tomography (CT), abdominal US, and bone scintigraphy to detect metastases. Because fluorodeoxyglucose positron emission tomography/computed tomography (FDG-PET/CT) can evaluate metastases in the entire body at one time, we have previously performed FDG-PET/CT to evaluate NAC efficacy. We inject [18F] 2-fluoro-2-deoxy-d-glucose (FDG), which is similar to glucose, into the body. We photograph the entire body with CT. So we detect cancerous lesions using the intrinsic property of cancer cells in which they uptake 3–8 times more glucose than normal cells. FDG-PET/CT has improved diagnostic strategies in cancer patients by identifying primary tumors and metastasis. A meta-analysis revealed that FDG-PET is a valuable tool for detecting breast cancer recurrence and metastases (Isasi et al. 2005). Some studies have reported that FDG-PET may become the method of choice for assessing asymptomatic patients with elevated tumor marker levels (Siggelkow et al. 2004). Moreover, FDG-PET/CT was reported to be useful as a prognostic indicator for patients with primary breast carcinoma and high standardized uptake value (SUV), which represented a worse prognosis with respect to both overall and relapse-free survival (Oshida et al. 1998).

As FDG-PET/CT has potential advantages over conventional imaging modalities, it may be useful for monitoring breast cancer treated with NAC. However, the effectiveness of FDG-PET/CT for evaluating NAC is controversial. For instance, Wahl et al. reported that FDG-PET of primary breast cancers showed a significant decrease in tumor glucose metabolism after effective treatment was initiated and tumor response was determined pathologically (Wahl et al. 1993). Tateishi et al. reported that the sensitivity of FDG-PET/CT for pCR was not acceptable, but the specificity was high (Tateishi et al. 2012). Therefore, herein we analyzed 83 breast cancer tumors with NAC treated from 2006. The purpose of this study was to evaluate whether FDG-PET/CT can determine NAC response and whether FDG-PET/CT can be a new prognostic marker by comparing FDG uptake or clinicopathologic characteristics before and after NAC.

## Results

### Clinicopathological characteristics

The clinical characteristics of the 83 breast cancers are summarized in Table 1. As two patients had breast cancer in both breasts, we analyzed 83 total tumors. All patients were female and the median age was 54 (age range 30–75 years). The histologic subtype was invasive ductal carcinoma in 79 (95.2 %), invasive lobular carcinoma in 1 (1.2 %), mucinous carcinoma in 1 (1.2 %), and apocrine carcinoma in 2 (2.4 %). Thirty-nine patients were treated with docetaxel followed by FEC (5-FU, epirubicin, and cyclophosphamide), thirty-nine patients were treated with weekly paclitaxel docetaxel followed by FEC, and five patients were treated with other regimens. One patient was treated with trastuzumab.

**Table 1:** Clinical characteristics of the patient cohort

	Number	%
Age		
Median	54	
Range	30–75	
Tumor size		
Median	30	
Range	13–120	
T status		
T1	8	9.6
T2	62	74.7
T3	7	8.4
T4	6	7.2
N status		
N0	40	48.2
N1	32	38.6
N2	7	8.4
N3	4	4.8
Stage before NAC		
Stage II	68	81.9
Stage III	15	18.1
Type of surgery		
Breast-conserving surgery	38	45.8
Mastectomy	36	43.4
Skin-sparing mastectomy	8	9.6
No operation	1	1.2
Type of axial surgery		
Sentinel lymph node biopsy	15	18.1
Axial dissection after sentinel lymph node biopsy	4	4.8
Axial dissection	63	75.9
No operation	1	1.2
Subtype		
Luminal A and luminal B	54	65.1
Luminal HER2	6	7.2
HER2 positive	10	12.0
Triple negative	13	15.7
Nuclear grade		
I	37	44.6
II	20	24.1
III	20	24.1
Unknown	6	7.2
FDG uptake		
Negative	0	0.0
Positive	83	100.0
Regimens of NAC		
wPAC → FEC	39	47.0
DOC → FEC	39	47.0
FEC → DOC	2	2.4
FEC → DOC + H	1	1.2
FEC → PTX + Bev	1	1.2
wPAC	1	1.2

*PAC* paclitaxel, *FEC* 5-FU, epirubicin and cycrophsphamide, *DOC* docetaxel, *H* trastuzumab, *Bev* bevacizumab

All patients except one underwent surgery: 38 breast-conserving surgeries with radiotherapy, 36 mastectomies, or eight skin-sparing mastectomies with breast reconstruction.

**Fig. 1:**
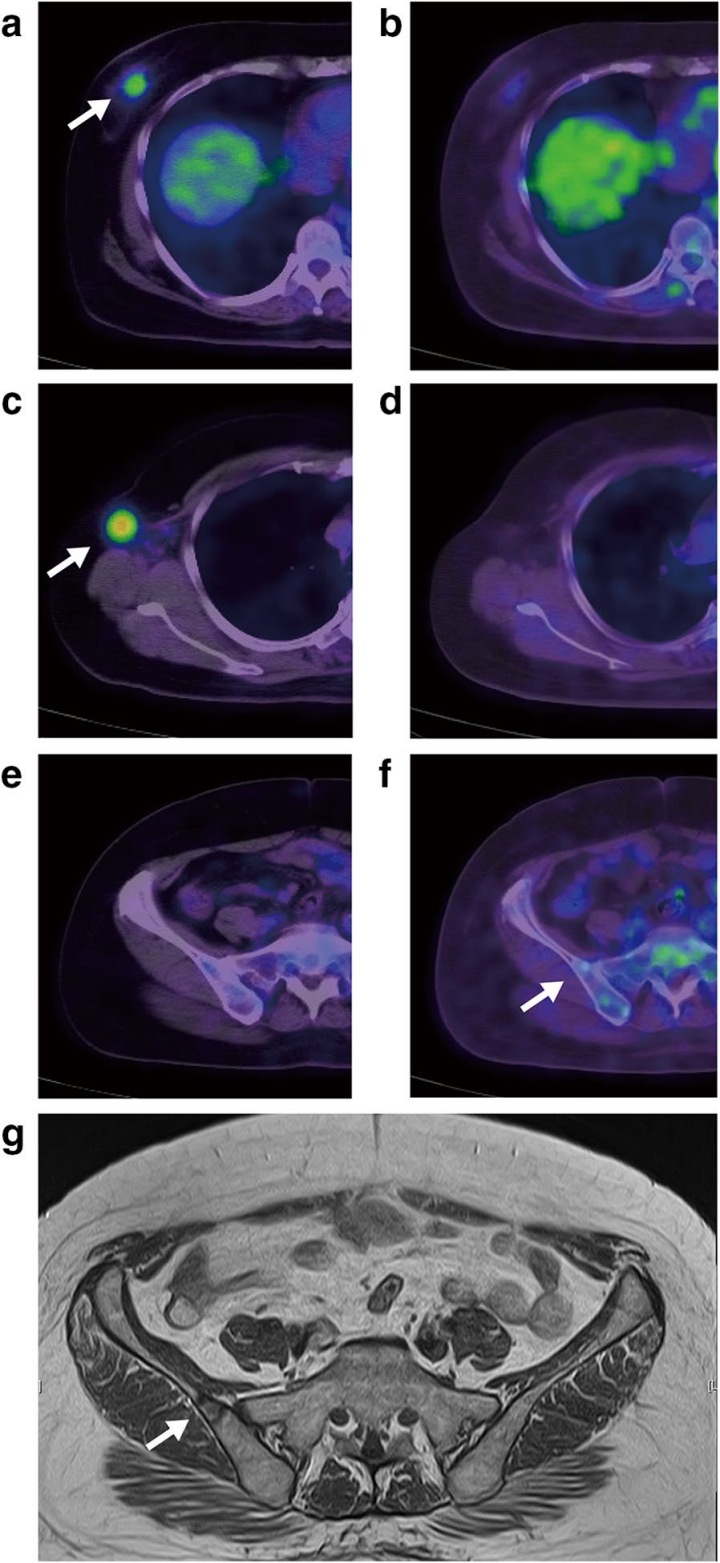
A 56-year-old woman with detected bone metastasis using FDG-PET/CT after NAC. **a**, **b** The abnormal FDG uptake of the primary lesion was 3.7 (**a**) before NAC and 1.4 (**b**) after NAC. **c**, **d** The abnormal FDG uptake of the axial lymph node was 6.1 (**c**) and disappeared after NAC. **e**, **f** Abnormal FDG uptake of the right ilium was not detected (**e**) before NAC and was 1.8 (**f**) after NAC. **g** Bone metastasis was varied in right ilium on MRI

After NAC, US, MRI, and FDG-PET/CT showed 16 (19.3 %) complete responses (CR), 54 (65.1 %) partial responses (PR), 11 (13.3 %) stable disease (SD), and 2 (2.4 %) progressive disease (PD). Two cases of PD were detected with bone metastases by FDG-PET/CT, even though the primary tumor shrunk after NAC (Fig. 1). One of the two patients did not undergo surgery and continued to be treated with chemotherapy. The other patient’s bone metastasis was doubted on FDG-PET after NAC.Though we suggested to her to continue chemotherapy, she strongly wanted to have a breast surgery done, so we did the surgery. Her bone matastasis was definitively diagnosed 6 months later. We experienced remote metastasis in 2.4 % of patients after NAC with FDG-PET/CT. The mean time interval from initial breast cancer diagnosis to first recurrence was 19 months (range 4–75 months). Overall, 23 patients (27.7 %) developed recurrence and 15 died from breast cancer during the follow-up period.

### FDG-PET/CT to predict pCR in breast cancer

**Fig. 2:**
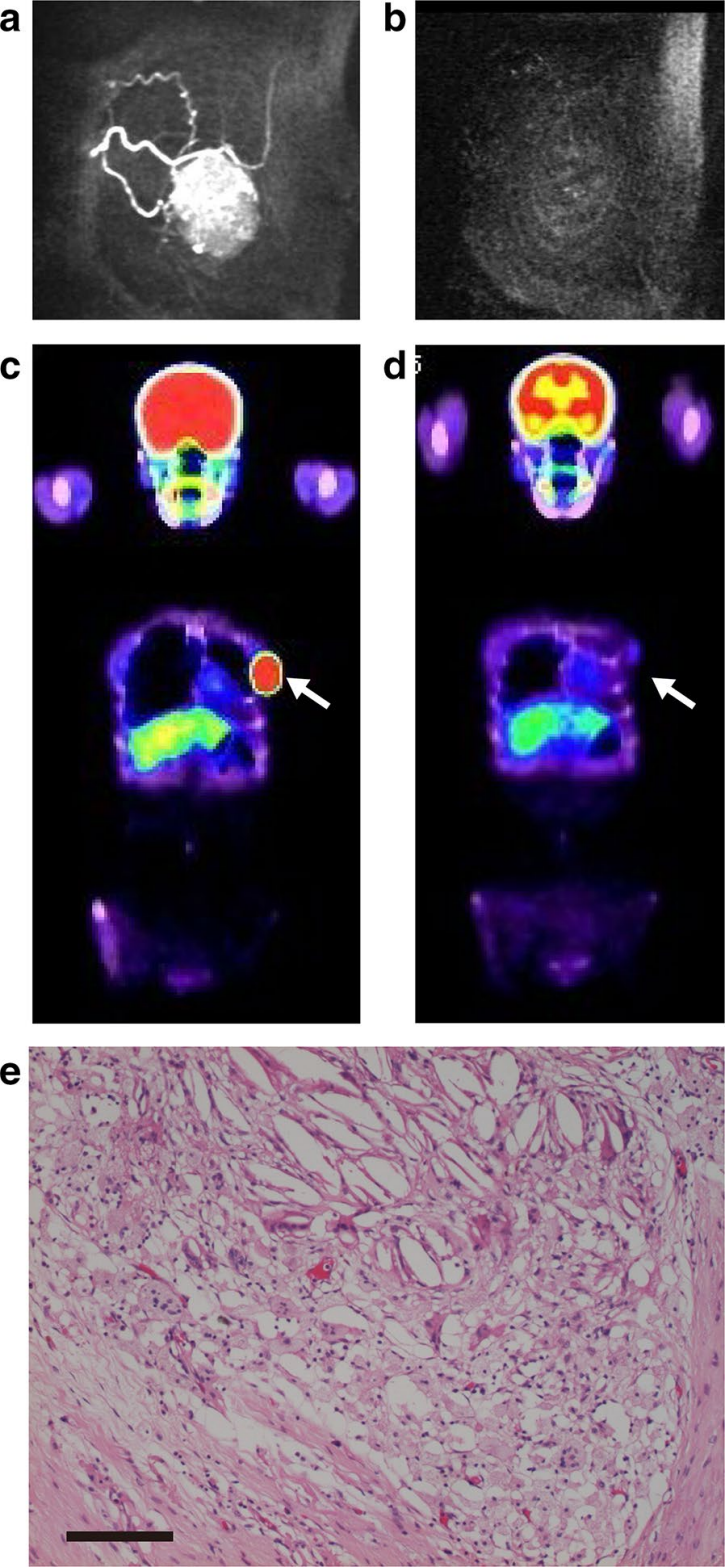
A 55-year-old woman had pCR with abnormal FDG uptake. **a** The cancer was visualized by a big mass on MRI before NAC. **b** MRI after NAC; the breast cancer could not be detected, which we considered as a complete clinical response. **c** FDG-PET/CT before NAC; the SUV_max_ of the primary breast tumor was 14.3. **d** FDG-PET/CT after NAC; abnormal FDG uptake remained (1.7). **e** Pathological findings: the cancer cells were gone. *Bar* 100 μm

In this study, we defined >1.7 SUV_max_ as abnormal FDG uptake and ypT0/is ypN0 as pCR in accordance with the method section. pCR was observed in 14 patients (16.9 %; Table 2). Estrogen receptor (ER) status and human epidermal growth factor receptor 2 (HER2) status were significantly associated with pCR. Abnormal FDG uptake after NAC might be associated with non-pCR, but this was not significant. Abnormal FDG uptake after NAC was analyzed in the two groups according to pathologic results (pCR and non-pCR). We evaluated the proposition that the abnormal uptake after NAC can predict the non-pCR. The sensitivity of that proposition was 20.3 % and the specificity was 94.7 % (Table 2). pCR with abnormal FDG uptake after NAC only occurred in one patient (Fig. 2). In this case, the reason for the abnormal FDG uptake was attributed to the fact that the primary tumor was too big and that macrophages and granulation tissue around the shrinking tumor were responsible for the FDG uptake. If the primary breast cancer doesn’t have abnormal FDG uptake accumulation after NAC, this does not indicate non-pCR due to low sensitivity; therefore, it is difficult to predict pCR with FDG-PET/CT. Thus, for predicting pCR, US and MRI may be more effective.

**Table 2:** A comparison of tumor responses

	PCR	Non-pCR	P values
preT			
T1	3	5	
T2	10	52	
T3	0	7	
T4	1	5	0.2755
Stage			
II	13	55	
III	1	14	0.2438
Estrogen receptor			
+	6	56	
−	8	13	0.0026*
HER2			
0	7	52	
1	1	5	
2	0	5	
3	6	7	0.0184*
PET			
<1.7	13	51	
>1.7	1	18	0.1240

* Statistically significant values

### Correlation between abnormal FDG uptake after NAC and prognosis

**Fig. 3:**
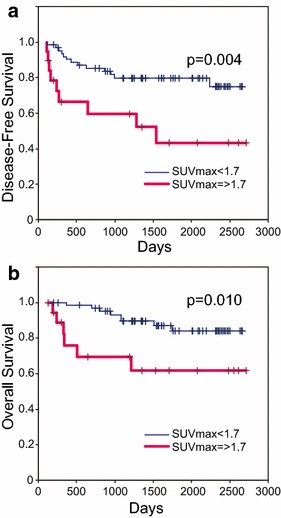
Kaplan–Meier curves of disease-free survival (**a**) and overall survival (**b**) according to SUV_max_

**Fig. 4:**
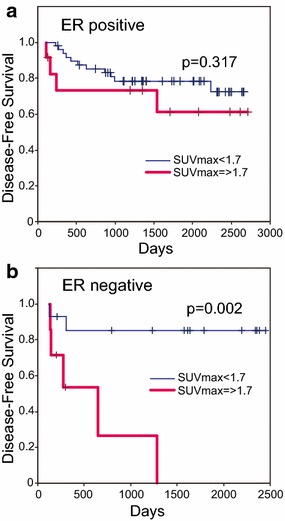
Kaplan–Meier curves of disease-free survival according to SUV_max_ in the ER-positive group (**a**) and the ER-negative group (**b**)

Next, we compared disease-free survival (DFS) and overall survival (OS) (Table 3). Among the clinicopathologic variables investigated, N stage, clinical stage, and abnormal FDG uptake after NAC were associated with DFS in the univariate analysis. Patients with the abnormal FDG uptake after NAC had significantly more recurrences (*P* = 0.004, Fig. 3a). We hypothesized that primary pCR would be associated with DFS, but we could not prove a significant association because of the small number of primary pCR cases. When multivariate analysis was performed with prognostic variables, N stage and abnormal FDG uptake after NAC were identified as significant independent prognosticator in this small subset of patients (N stage hazard ratio: 4.316, *P* = 0.010; abnormal FDG uptake after NAC hazard ratio: 2.955, *P* = 0.014). As for OS, clinical N stage, clinical stage, and abnormal FDG uptake after NAC were associated with OS in the univariate analysis among the clinicopathologic variables investigated. More patients with abnormal FDG uptake after NAC died (*P* = 0.010, Fig. 3b). When multivariate analysis was performed with prognostic variables, only abnormal FDG uptake after NAC was a significant independent prognosticator (hazard ratio: 2.643, *P* = 0.029). As shown in Table 2, the tumor response was significantly different between ER-positive and ER-negative tumors. Comparing DFS of ER-positive and ER-negative patients, this significant difference disappeared in the ER-positive group and the significant difference was greater in the ER-negative group (Fig. 4).

**Table 3:** The relationship between clinicopathologic factors and prognosis

	n	DFS			OS		
		Univariate analysis	Multivariate analysis		Univariate analysis	Multivariate analysis	
		P value	Hazard ratio	P value	P value	Hazard ratio	P value
Age							
≤45	15	0.858			0.219		
>45	67						
T							
T1/2	69	0.078			0.112		
T3/4	13						
N							
0	40	0.001*	4.316	0.010*	0.026*	2.927	0.113
1/2/3	42						
Stage							
II	67	0.037*	1.762	0.229	0.037	2.278	0.154
III	15						
ER							
+	63	0.858			0.524		
−	19						
HER2							
0, 1	65	0.985			0.734		
2, 3	17						
pCR							
+	17	0.388			0.996		
−	65						
pSUV							
<1.7	63	0.004*	2.955	0.014*	0.010*	2.643	0.029*
>1.7	19						

* Statistically significant values

## Discussion

In this study, we analyzed FDG-PET/CT after NAC in breast cancer tumors. The clinical benefit of FDG-PET/CT includes the detection of the original lesion and metastasis to axillary lymph nodes, diagnosis of remote metastases, treatment effect measurement, and convalescence prediction (Minamimoto et al. 2007). FDG-PET/CT has also been applied to assess the stage of malignant viability as well as to monitor treatment response.

It is well known that breast cancer is a heterogeneous disease. Classification according to clinical subtype with ER and HER2 gained widespread acceptance and these tumors have been shown to differ in presentation, response, to treatment and prognosis (Straver et al. 2010). Therefore, we must also accommodate for heterogeneity in treatment and assessment of breast cancer.

In this study, we set an SUV_max_ cutoff of 1.7. With respect to FDG-PET/CT, how to evaluate SUV_max_ is controversial. Some researchers have assessed SUV as the SUV reduction rate, response rate, or delta SUV (Connolly et al. 2015; Jung et al. 2010; Choi et al. 2010). Indeed, the reduction or difference in SUV before and after NAC may exactly correlate with tumor response. However, breast cancer has such heterogeneity that some tumors exhibit high SUV, while others show low initial SUV. Therefore, we examined the SUV minimum of the abnormal FDG uptake in breast cancer and used a cut-off of 1.7.

We showed that FDG-PET/CT is useful for predicting prognosis in our cohort; cases with abnormal FDG uptake had a significantly worse prognosis. A similar result was generated by the multivariate analysis. Some reported reduction of FDG uptake could be used as a predictive marker for DFS, and FDG uptake for 2 cycles of NAC was associated with shorter event-free survival with ER +/HER2- breast cancer (Jung et al. 2010; Groheux et al. 2015).

Further in our study, as the significant difference disappeared in the ER-positive group on multivariate analysis, FDG uptake may represent poor-prognosis in the ER-negative group. Even if the NAC effect is not evident in the ER-positive group, postoperative hormone therapy is often effective. This may be the reason why abnormal FDG uptake doesn’t necessarily indicate a poor prognosis. Some reported FDG-PET/CT findings may contribute to differentiation of luminal A and non-luminal A subtypes of invasive breast cancer (Miyake et al. 2014). Moreover, FDG-PET/CT response may differ in ER-positive and ER-negative tumors. In this study, we only evaluated the difference in ER-positive and ER-negative tumors. Some researchers have reported that FDG-PET/CT during NAC may predict response in ER-positive/HER2-negative and triple negative tumors, but not in HER2-positive breast cancer (Koolen et al. 2013). Therefore, in the future, we want to evaluate differences according to subtype with more patients and a longer follow-up time.

Breast cancer bone metastases are harder to detect than liver or pulmonary metastasis with CT and US. Ohta compared 99Tcm-methylene diphosphonate bone scintigraphy with FDG-PET for diagnosing breast cancer bone metastasis, and determined a sensitivity of 77.7 % and specificity of 97.6 % for the latter, while the sensitivity of bone scintigraphy was 77.7 % and specificity was 80.9 % (Ohta et al. 2001). The sensitivity of osteoplastic and osteoclastic metastases are equal using both imaging modalities. However, the specificity of FDG-PET/CT was significantly better than bone scintigraphy (*P* = 0.0392); therefore, FDG-PET/CT can be considered a substitute for bone scintigraphy. Because FDG-PET/CT can evaluate metastases in the whole body at one time, FDG-PET is useful for broad metastasis investigations in breast cancer patients. In the present study, we detected remote metastasis after NAC in 2.4 % of patients.

Caudle et al. reported 59 cases with PD of 1928 (3 %) NAC-treated patients, but remote metastasis was evident in only three of 1928 patients (0.015 %) (Caudle et al. 2011). In our study, the remote metastasis frequency was 2.4 %. Caudle et al. did not mention the modality used to generate the remote metastasis diagnosis, but our detection frequency was higher than they reported.

## Conclusion

We examined the accuracy of FDG-PET after NAC for detecting metastases. FDG-PET after NAC was not suitable for treatment efficacy evaluation, but it was effective for predicting prognosis. Moreover, this tendency was particularly remarkable in ER-negative breast cancer. In addition, we determined a remote metastasis rate of 2.6 % after NAC, and in these cases we changed the treatment protocol to avoid surgery. As this study was retrospective and the number of the patients was small, the results of our study should be interpreted with causion. In conclusion, FDG-PET/CT is useful for predicting the prognosis in NAC-received breast cancer patietnts and reevaluating operation applicability after NAC.

## Methods

### Ethics statement

This study was performed in accordance with the guidelines approved by the Ethics Committee of Tokyo Medical and Dental University (TMDU) and the Declaration of Helsinki for biomedical research involving human subjects, and was approved by The Institutional Review Board of TMDU. All patients had breast cancer and consented to NAC and FDG-PET/CT. We contacted the patients and got the written informed consent of this study. But as we could not obtain some patients’ written informed consent, the information of this study was posted in the hospital and on the website.

### Patients

A retrospective analysis was performed on 81 consecutive breast cancer patients who were treated with NAC at our institution from September 2006 to September 2013. As two patients had cancer in both breasts, we analyzed 83 total breast cancer tumors. All patients were diagnosed with stage II or III breast cancer and underwent NAC, including a patient who was treated with NAC, but not surgery, because of progression during NAC. We observed these patients for a median of 50 months.

### Clinicopathological evaluations

Baseline examinations were performed before the first cycle of chemotherapy and included a physical examination, mammography, US, enhanced MRI, and FDG-PET/CT. Lymph node metastases were determined by US, MRI, PET, and fine-needle aspiration cytology. ER and progesterone receptor (PgR) status were evaluated by J-score. The J-score comprises proportional values irrespective of the intensity of stained nuclei, and the proportion of cells stained in each specimen was recorded as 0, none; 1, <1 %; 2, 1–10 %; 3, ≥10 %, as advocated and employed as cut-off points in previous reports (Umemura et al. 2006). HER-2 positivity was defined by immunohistochemistry. After completion of NAC, clinical response was assessed using the Response Evaluation Criteria in Solid Tumors 1.1 by US and MRI. The exact definition of pCR in breast cancer is unclear (von Minckwitz et al. 2012). We adopted a pCR of ypT0/is ypN0, which means no invasive residual cancer in the breast and no infiltrated lymph node, but noninvasive breast residuals permitted.

### NAC and surgery

NAC regimens consisted of both four cycles of an anthracycline regimen and four cycles of a taxane regimen. Only one patient was treated with trastuzumab. After NAC completion, all patients except the patient with detected metastasis underwent either breast-conserving surgery with radiotherapy, mastectomy, or skin-sparing mastectomy with breast reconstruction. Breast reconstruction was performed by a plastic surgeon. The decision to operate was made following discussion between the surgeon and patient.

### Fdg-PET/CT

PET/CT images were acquired using a PET/CT system (Aquiduo; Toshiba Medical Systems, Tokyo, Japan) combining a full-ring PET scanner and a 16-row helical CT scanner. The maximal standardized uptake value (SUV_max_) was used to evaluate FDG uptake in the primary breast cancer. SUV_max_ was calculated as follows: SUV_max_ = measured activity concentration [Bq/mL]/(injected activity [Bq]/body weight [kg] × 1000). Blood glucose levels were measured just before FDG injection to exclude hyperglycemic patients whose serum glucose concentrations exceeded 200 mg/dL. After fasting for at least 4 h, the patients received an intravenous injection of 18F-FDG (3.7 MBq/kg). Visual assessment of 18F-FDG uptake was determined by consensus by two radiologists (Machida et al. 2013).

### Determination of the cutoff value for FDG-PET/CT

As we previously analyzed 110 breast cancers with FDG PET/CT, breast cancer tumors that were smaller than 2 cm and with an SUV_max_ <1.7, were all early stage breast cancers, such as either pN0, no lymph-vascular invasion, or low nuclear grade. Therefore, we defined >1.7 SUV_max_ as abnormal FDG uptake in this study.

### Statistical analysis

The Chi square test was used to evaluate clinicopathological characteristics. Kaplan–Meier test was used to evaluate DFS and OS in the univariate analysis. Cox proportional hazards regression analysis was used to evaluate the prognosis in the multivariate analysis. *P* values <0.05 were considered to indicate statistically significant differences. All statistical analyses were performed using the SPSS software (IBM Corp, Armonk, NY, USA).

## Abbreviations

NAC: neoadjuvant chemotherapy
pCR: pathological complete response
US: ultrasound
MRI: magnetic resonance imaging
CT: computed tomography
FDG-PET/CT: fluorodeoxyglucose positron emission tomography/computed tomography
FDG: [18F] 2-fluoro-2-deoxy-d-glucose
SUV: standardized uptake value
FEC: 5-FU, epirubicin, and cyclophosphamide
CR: complete responses
PR: partial responses
SD: stable disease
PD: progressive disease
ER: estrogen receptor
HER2: human epidermal growth factor receptor 2
DFS: disease-free survival
OS: verall survival
TMDU: Tokyo Medical and Dental University
PgR: progesterone receptor
